# Fabrication and Characterization of High Internal Phase Pickering Emulsion Gels Stabilized by Hesperidin and Lysozyme

**DOI:** 10.3390/foods15101636

**Published:** 2026-05-08

**Authors:** Xiaohong Ge, Yuxiang Wang, Guoyang Liu, Benguo Liu, Sheng Geng

**Affiliations:** School of Food Science, Henan Institute of Science and Technology, Xinxiang 453003, China; gexh801210@163.com (X.G.); 15738523872@163.com (Y.W.); liuguoyang@stu.hist.edu.cn (G.L.); liubenguo@hist.edu.cn (B.L.)

**Keywords:** citrus flavonoids, lysozyme, high internal phase Pickering emulsions, emulsifying properties

## Abstract

The development of novel multifunctional emulsifiers using protein–polyphenol interactions is a common strategy. Previously, we investigated the emulsifying properties of the four citrus flavonoids alone. This study investigated how complexing lysozyme (LY) with four citrus-derived flavonoids affects emulsifying properties. Results demonstrated that the emulsification performance was enhanced when flavonoids were complexed with LY, following the order: hesperidin (Hpd) > neohesperidin dihydrochalcone (Neohpddic) > neohesperidin (Neohpd) > hesperetin (Hpt). This enhancement was positively correlated with the intrinsic emulsification abilities of the flavonoids, suggesting that the synergistic effect should not overlook the emulsifying capacity of the flavonoids themselves. The Hpd-LY mixture increased the three-phase contact angle (to near 90°) compared to Hpd alone (51.16° ± 0.58), which helped form high internal phase emulsion (HIPE) gels. Stable HIPEs were achieved at an oil phase fraction φ = 80%, mixture concentration w ≥ 0.8%, and Hpd-to-LY ratio k ≥ 1:1. Droplet size decreased as w increased from 0.6% to 1.2%, but increased with higher φ and k, while gel strength improved. In addition, these HIPEs protected encapsulated lutein and suppressed lipid oxidation. The findings show that flavonoid–protein complexes, especially Hpd-LY, can build stable and functional HIPEs for protecting bioactive compounds.

## 1. Introduction

Citrus flavonoids, a class of naturally occurring polyphenolic compounds predominantly found in citrus fruits, include hesperetin (Hpt), hesperidin (Hpd), and neohesperidin (Neohpd). These compounds, characterized by their distinctive chemical structures, exhibit a wide array of biological activities, including antioxidant, anti-inflammatory, antiviral, anticancer, and metabolic regulatory effects. The health benefits associated with citrus flavonoids have attracted considerable interest within the domains of functional foods and health sciences [1]. In practical applications, citrus flavonoids encounter significant challenges, including poor water solubility, low bioavailability, and insufficient stability [2]. These limitations substantially hinder their widespread utilization in food and pharmaceutical products. As a result, recent research has increasingly shifted focus from exploring their chemical properties and biological functions to developing technological strategies that enhance their stability, solubility, and absorption efficiency within the body. To address the delivery and application of citrus flavonoids, researchers have proposed various advanced carrier systems. Among these, emulsion-based delivery systems have emerged as a pivotal approach due to their highly tunable structures, adjustable interfacial properties, and robust stability [3]. Notably, Pickering emulsions, which are surfactant-free and exhibit strong interfacial stability, demonstrate promising potential for the effective delivery of citrus flavonoids [4].

Protein–polyphenol complexes have been extensively studied for their potential as novel multifunctional emulsifiers. These complexes exhibit multiple functionalities, including antioxidant activity, nutritional enhancement, and color modulation, while demonstrating superior performance in both the formation and stabilization of emulsions [5]. Research suggests that protein–polyphenol complexes possess a higher molecular density at the oil–water interface compared to individual polyphenols, enhancing both antioxidant properties and the physical barrier effect [6]. Some researchers have proposed that the binding of proteins with non-polar polyphenols increases the surface hydrophobicity of the resulting complexes by introducing numerous hydrophobic groups, thereby improving their emulsifying properties compared to native proteins [7]. For instance, Zou et al. [8] modified the hydrophobicity of zein through complexation with tannic acid, resulting in composite particles capable of stabilizing the rheological behavior of emulsion gels. Similarly, the interaction between bovine β-lactoglobulin and procyanidins has been shown to improve the storage stability of emulsions [9]. Moreover, protein–polyphenol complexes serve as effective shell materials for encapsulating oxygen-sensitive components, with their antioxidant properties and functional activity contributing to the enhanced performance of emulsions.

Lysozyme (LY) is a natural antibacterial protein with some emulsifying properties. However, it is challenging for LY to effectively stabilize high internal phase Pickering emulsions (HIPEs) when used alone [10]. Recent research has demonstrated that LY can synergistically form HIPEs when combined with emulsifying flavonoids such as dihydromyricetin [11]. This combination not only reduces the quantity of flavonoids required but also enhances the stability of the emulsions. Additionally, recent studies have identified certain citrus flavonoids capable of directly stabilizing Pickering emulsions [12]. Furthermore, these flavonoids can facilitate the formation of Pickering emulsions through interactions with proteins, polysaccharides, or other functional particles, thereby improving the dispersibility, oxidative stability, and intestinal absorption of encapsulated compounds [13].

The emulsifying properties of the citrus flavonoids used in this study have been systematically characterized by our group [12]. Notably, in addition to being bioactive compounds that require efficient delivery, citrus flavonoids themselves can also act as functional emulsifiers, endowing the emulsion system with antioxidant and other biological activities. Consequently, flavonoid-stabilized emulsions themselves become excellent delivery vehicles for encapsulating and protecting other hydrophobic nutrients. However, in previous studies on flavonoid–protein complexes, the enhanced emulsifying performance has often been simply attributed to changes in the surface hydrophobicity of the protein, while the possible contribution from the intrinsic emulsifying ability of the flavonoids themselves has been largely overlooked. A key question therefore arises: is the improvement in emulsification after mixing correlated with the self-emulsifying property of the flavonoids? If yes, the common practice of attributing the enhanced emulsification solely to protein surface hydrophobicity changes would need to be re-examined. Therefore, this study systematically investigates the emulsification behavior of four citrus-derived flavonoids—Hpt, Hpd, Neohpd, and neohesperidin dihydrochalcone (Neohpddic)—when combined with LY. By assessing emulsification performance, interfacial properties, and emulsion stability, the research aims to identify the optimal flavonoid–protein combination. Additionally, the study explores the impact of oil phase volume fraction (φ), mixture concentration (w), and Hpd-to-LY ratio (k) on emulsion structure, lutein protection capability, and anti-lipid oxidation performance. This research seeks to provide a theoretical foundation and technical support for the use of flavonoid–protein composite systems in developing green, functional emulsions.

## 2. Materials and Methods

### 2.1. Chemicals

Hesperetin (Hpt), hesperidin (Hpd), neohesperidin (Neohpd), neohesperidin dihydrochalcone (Neohpddic), lysozyme (LY) (all ≥98% purity), and food-grade medium-chain triglycerides (MCT) were supplied by Shanghai Yuanye Bio-Technology Co., Ltd. (Shanghai, China). Food-grade sunflower oil was obtained from Yihai Kerry Grain and Oil Food Industrial Co., Ltd. (Hefei, China). Lutein and all fluorescent dyes (Nile Blue A and Nile Red) were sourced from Shanghai Aladdin Biochemical Technology Co., Ltd. (Shanghai, China) and Sigma-Aldrich (St. Louis, MO, USA), respectively. Ultra-pure water was obtained using a GenPure UV/UF system (Thermo Fisher Scientific, Waltham, MA, USA). All other reagents were of analytical grade.

### 2.2. Procedure for Evaluation of Emulsion Gel Formation

Appropriate proportions of LY were combined with Hpt, Hpd, Neohpd, or Neohpddic, and the mixtures were dissolved in the aqueous phase. MCT served as the oil phase. The two phases were homogenized using an Ultra-Turrax T18 disperser (IKA, Staufen, Germany) at 8000 rpm for 3 min. Pickering emulsions with different oil phase volume fractions (φ = 80% and 50%) were prepared by varying the mass ratios of each flavonoid to LY (w = 0.8%, k = 4:1, 2:1, 1:1, 1:2, 1:4). After preparation, the emulsions were allowed to stand for 24 h. Emulsion gel formation was evaluated by the tube inversion method: samples that did not flow upon inversion for 2 h were considered stable emulsion gels [14,15].

### 2.3. Measurement of Contact Angle

The three-phase contact angle (θ) was measured to evaluate the wettability of the samples, using an optical tensiometer (Theta Lite, Biolin Scientific, Stockholm, Sweden) and the sessile drop technique. The samples for measurement consisted of Hpd and LY pre-mixed at a 1:1 mass ratio. Cylindrical tablets were first prepared by compressing the sample powders under 15 MPa pressure. Each tablet was then placed in a cubic quartz container (Lianyungang Shengfan Quartz Product Co., Ltd., Lianyungang, China) (with 5 cm sides) filled with MCT. A 25 µL water droplet was carefully dispensed onto the tablet surface using a high-precision automated dispenser (Theta Lite, Biolin Scientific, Stockholm, Sweden). The droplet profile was recorded immediately for 15 s, and θ was calculated by applying an elliptical fitting model [16].

### 2.4. Preparation of the Emulsion

The emulsions were prepared according to the method described in Section 2.2 to systematically investigate the effects of the oil phase volume fraction (φ = 50%, 60%, 70%, 80%, 90%), total mixture concentration (w = 0.4%, 0.6%, 0.8%, 1.0%, 1.2%), and Hpd-to-LY mass ratio (k = 5:1, 3:1, 1:1, 1:3, 1:5) on the formation of Pickering emulsion gels [17,18].

### 2.5. Fluorescence Spectroscopy Measurement

A stock solution of LY (1 mg/mL) was prepared by dissolving 0.1 g of the protein in phosphate buffer (10 mM, pH 7.0). The flavonoids (Hpt, Hpd, Neohpd, Neohpddic) were first dissolved in a small volume of DMSO and then diluted with phosphate buffer to achieve the desired concentrations, ensuring the final DMSO content did not exceed 1%. Then, 4 mL of the LY solution was mixed with 1 mL of each flavonoid solution at varying concentrations (3, 6, 9, 12, 15, and 18 μmol/L). The mixtures were incubated in the dark at 303 K for 30 min. Following incubation, fluorescence emission spectra were recorded from 300 to 450 nm using a Cary Eclipse G9800A spectrophotometer (Agilent Technologies, Santa Clara, CA, USA), with an excitation wavelength of 280 nm. For all measurements, the excitation and emission slit widths were set at 5 nm, and the detector voltage was maintained at 680 V [19].

### 2.6. Determination of Droplet Diameter in HIPEs

Emulsions were prepared with varying parameters to study their effects on droplet diameter. The tested variables and their ranges were as follows: w (0.6%, 0.8%, 1.0%, 1.2%; at φ = 80% and k = 3:1), φ (50%, 60%, 70%, 80%; at w = 0.8% and k = 3:1), and k (5:1, 3:1, 1:1, 1:3; at w = 0.6% and φ = 80%). Droplet size distribution was measured using a BT-9300H laser particle size analyzer (Bettersize Instruments Ltd., Dandong, China) with water as the dispersant. Data acquisition was initiated once the sample obscuration reached 15–16% [20].

### 2.7. Determination of Emulsion Type

Emulsion types were identified by visualizing the distribution of fluorescent dyes with an LSM780 laser scanning confocal microscope (CLSM; Carl Zeiss, Baden-Württemberg, Germany). For this purpose, the aqueous phase was pre-stained with a 0.1 mg/mL solution of Nile Blue A, while the oil phase was pre-labeled with a 0.01% Nile Red solution prepared in 1,2-propanediol. The dyes were excited at 488 nm (Nile Red) and 633 nm (Nile Blue A). Representative emulsions, formulated under two distinct sets of conditions (φ = 80%, w = 1.0%, k = 5:1 and φ = 80%, w = 1.2%, k = 3:1), were subjected to imaging. Images were acquired at a resolution of 1024 × 1024 pixels and subsequently processed with ZEN 3.0 software [21]. In the resulting CLSM images, the color channels were swapped (blue for oil, red for particles) to improve visual contrast against background fluorescence.

### 2.8. Determination of Textural Properties of HIPEs

The textural properties (specifically gel strength) of Pickering emulsions prepared with different w and φ values were measured using a texture analyzer (A-XT Plus, Stable Micro Systems Ltd., Surrey, UK). The instrument was equipped with a P/0.5 probe and operated using the Gelatin Gel Strength Measurement mode. The pre-test, test, and post-test speeds were all set to 1.0 mm/s. Measurements were performed with a trigger force of 3.0 g and a penetration distance of 4 mm [22].

### 2.9. Determination of the Protective Effect of HIPE Lutein

To assess the protective capacity of the Hpd-LY HIPE gels for hydrophobic bioactives (including flavonoids), lutein was used as a model photosensitive nutrient. HIPEs were prepared using MCT oil containing 0.5 mg/mL lutein. To investigate the effect of mixture concentration, emulsions were prepared with w set at 0.8%, 1.0%, and 1.2%, while maintaining a constant φ of 80% and a k of 1:1. To examine the effect of the Hpd-to-LY ratio, emulsions were prepared with k at 3:1, 1:1, and 1:3, while keeping w at 1.0% and φ at 80%. The prepared emulsions were incubated at 37 °C and subjected to UV light to accelerate lutein degradation. Daily, a 50 mg sample of emulsion was collected and mixed with 6 mL of an n-hexane/ethanol mixture (2:1, *v*/*v*) and 1 mL of water. The mixture was vortexed for 30 s and then centrifuged at 2000× g for 6 min. After allowing the samples to stand in the dark for 10 min, the absorbance of the supernatant was measured at 450 nm. The protective effect of the HIPEs on lutein was evaluated by comparing the lutein retention rates against control groups, which consisted of lutein dissolved in plain MCT oil and emulsions stabilized with Tween 80 [23].

### 2.10. Determination of Lipid Oxidation by HIPEs

Following the method of Wang et al. [23], the hydrogen peroxide content in the emulsions was measured during incubation at 37 °C. At predetermined time points, a 50 mg sample was taken for analysis. An emulsion stabilized with Tween 80 was used as a control.

### 2.11. Statistical Analysis

Data are presented as the mean ± standard deviation of triplicate measurements. Differences among groups were assessed by one-way analysis of variance (ANOVA) followed by Duncan’s multiple range test. A probability value of *p* < 0.05 was considered statistically significant.

## 3. Results and Analysis

### 3.1. Evaluation of Emulsion Gel Formation

**Figure 1:**
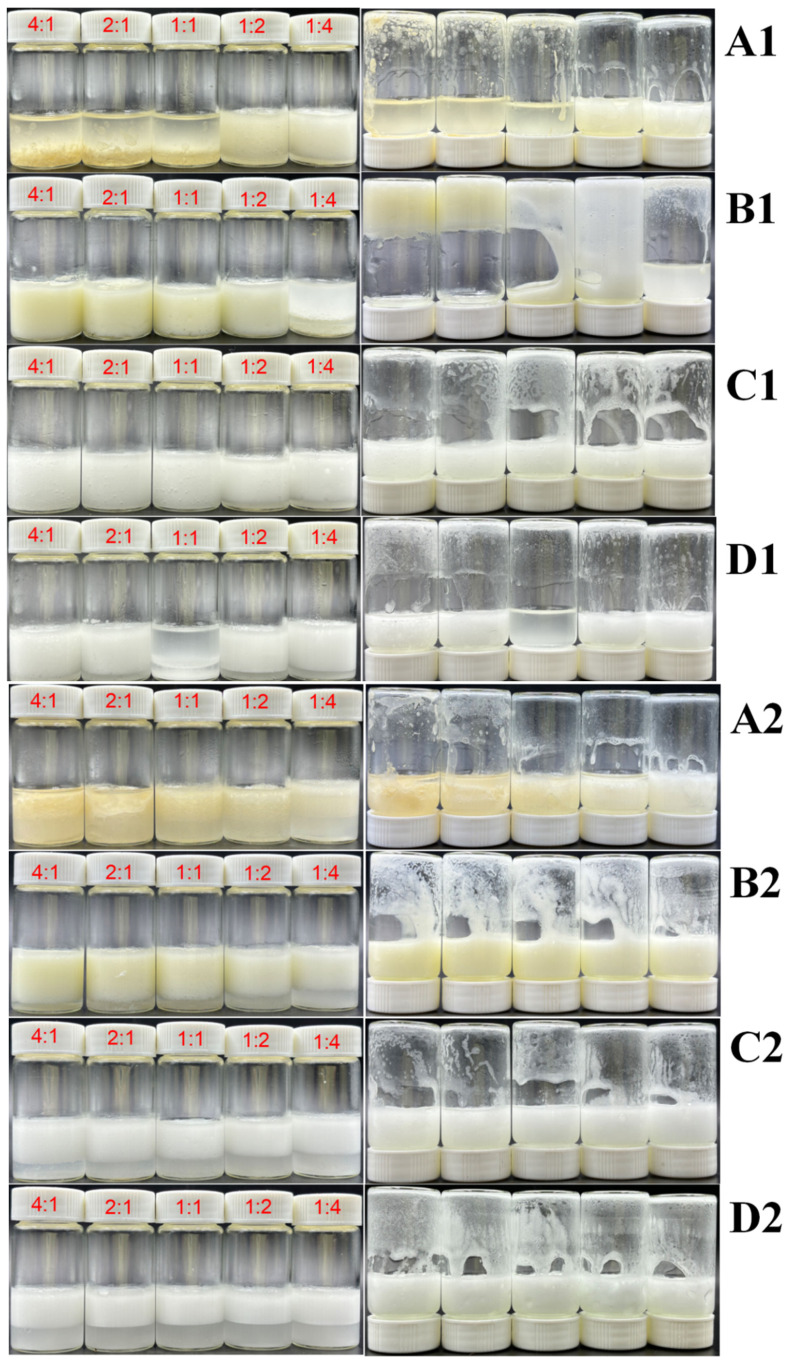
The effect of the mass ratio of Hpt, Hpd, Neohpd, Neohpddic to LY on the emulsion. ((**A1**–**D1**): w = 0.8%, φ = 80%, from left to right, the mass ratio k = 4:1, 2:1, 1:1, 1:2, 1:4; (**A2**–**D2**): w = 0.8%, φ = 50%, from left to right, the mass ratio k = 4:1, 2:1, 1:1, 1:2, 1:4. In (**A1**–**D2**), the (**left side**) shows the state after 24 h of standing, and the (**right side**) shows the state after 2 h of inversion).

The emulsifying performance of four flavonoid-LY complexes—Hpt, Hpd, Neohpd, and Neohpddic—was assessed across different mass ratios and two oil phase fractions: high (φ = 80%) and low (φ = 50%). As illustrated in Figure 1, after stabilizing the emulsions for 24 h, emulsion gel formation was evaluated by the tube inversion method. The inversion method demonstrated that combinations of LY with Hpt, Neohpd, and Neohpddic did not yield stable emulsion gels in either high or low oil phase conditions. This indicates that the addition of LY does not significantly enhance emulsification capacity. Conversely, when the φ is 80% and the mass proportion of Hpd exceeds 50%, stable emulsion gels can be formed even at a lower addition level (w = 0.8%) (Figure 1(B1)). However, at φ = 50%, stable emulsion gels are not formed (Figure 1(B2)). These findings suggest that the combination of Hpd with LY effectively stabilizes HIPEs, likely due to Hpd’s superior emulsifying capacity compared to the other flavonoids, which enables enhanced synergistic effects when combined with LY. This phenomenon may be related to the enhanced interfacial adsorption and surface hydrophobicity of protein–polyphenol complexes, which promote the formation of a dense interfacial layer around oil droplets and thereby improve emulsion stability [24]. Consequently, given Hpd’s robust emulsifying ability, it is selected as the emulsifier for further emulsion gel research.

### 3.2. Fluorescence Spectroscopic Analysis

**Figure 2:**
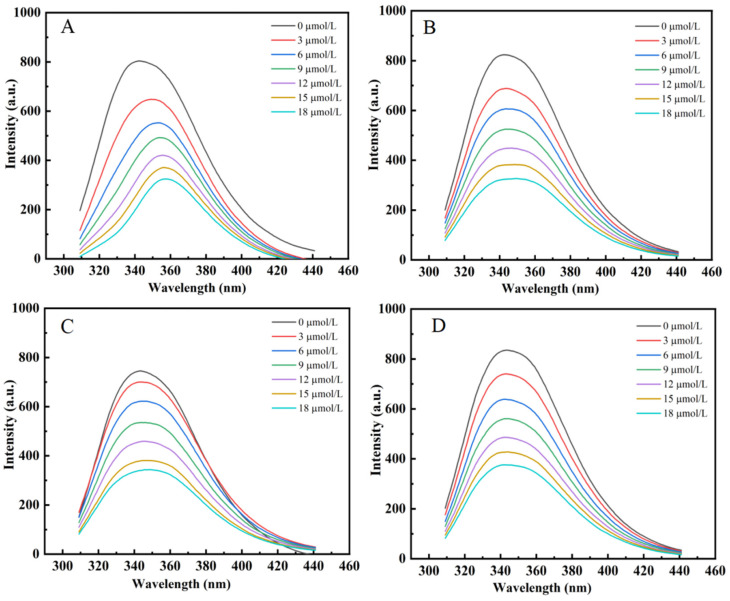
Effect of flavonoids on LY fluorescence spectra ((**A**): Hpt; (**B**): Hpd; (**C**): Neohpd; (**D**): Neohpddic).

Owing to the presence of intrinsic fluorophores—tryptophan, tyrosine, and phenylalanine—LY displays characteristic fluorescence, a property common to many fluorescent proteins [25]. The present work investigated how four structurally analogous citrus flavonoids modulate the fluorescence spectrum of LY (refer to Figure 2). Upon excitation at 280 nm, LY displayed a characteristic emission maximum at 341 nm. A systematic and significant reduction in the fluorescence intensity of LY was observed with increasing flavonoid concentration, demonstrating that all four compounds act as fluorescence quenchers. The quenching efficacy, however, varied considerably, with Hpd inducing the most pronounced suppression, followed by Neohpddic, Neohpd, and Hpt being the least effective. These findings suggest interactions between the flavonoids and LY, and imply that the presence of glycosyl substituents in flavonoid molecules may influence their binding mode with the protein, thereby affecting the degree of fluorescence quenching [26]. Previous studies have shown that fluorescence quenching in flavonoid–protein systems is predominantly static in nature and is primarily driven by hydrogen bonding, hydrophobic interactions, and van der Waals forces. The type and distribution of substituent groups on the flavonoid skeleton are key determinants of binding affinity. Yang et al. have pointed out that the number of hydrogen atoms and the position of glycosyl substitution significantly influence binding [27], while Meng et al. further demonstrate that structural variations lead to differences in the dominant interaction forces and binding affinities [28]. Moreover, in bovine serum albumin and α-glucosidase systems, flavonoids typically bind within hydrophobic pockets of the protein and perturb the microenvironments of tryptophan and tyrosine residues, thereby inducing fluorescence quenching; the binding strength correlates closely with the number of hydroxyl groups and the degree of glycosylation [29,30]. Consequently, Hpd exhibits the strongest quenching capacity, likely due to its greater propensity to form stable complexes with LY and to access the chromophores more readily, whereas the glycosylated flavonoids Neohpd, Neohesperidin, and Hpt show weaker quenching, which may be attributed to increased steric hindrance and altered polarity that diminish their interaction strength with the protein.

### 3.3. Wettability of Particles

The θ serves as a key indicator for evaluating the wettability characteristics of solid particle surfaces. Particles with optimal wettability exhibit strong adsorption at the oil–water interface. This adsorption creates a steric hindrance effect, which serves to inhibit droplet coalescence and improve emulsion stability [31]. As depicted in Table 1, the θ of Hpd at the oil–water interface is relatively small, indicating its relatively strong hydrophilicity. In Pickering emulsion stabilization, a θ close to 90° is considered optimal, as it maximizes emulsion stability. The contact angle of LY is closer to 90°, indicating that LY possesses more ideal wettability characteristics [32]. When k = 1:1 ratio, the contact angle of the mixture increases compared to Hpd alone, suggesting altered wettability characterized by increased hydrophobicity. This change may result from interactions between Hpd and LY that enhance the particles’ affinity for the oil phase, thereby boosting their potential to stabilize Pickering emulsions. This finding is consistent with the emulsification capacity results in Section 3.1, providing additional evidence that the Hpd-LY combination effectively promotes Pickering emulsion stability. Similar results have been reported that particles with a contact angle close to 90° exhibit balanced wettability toward oil and water phases, enabling strong adsorption at the oil–water interface and leading to enhanced Pickering emulsion stability [33].

### 3.4. Formation of Emulsion Gels

**Figure 3:**
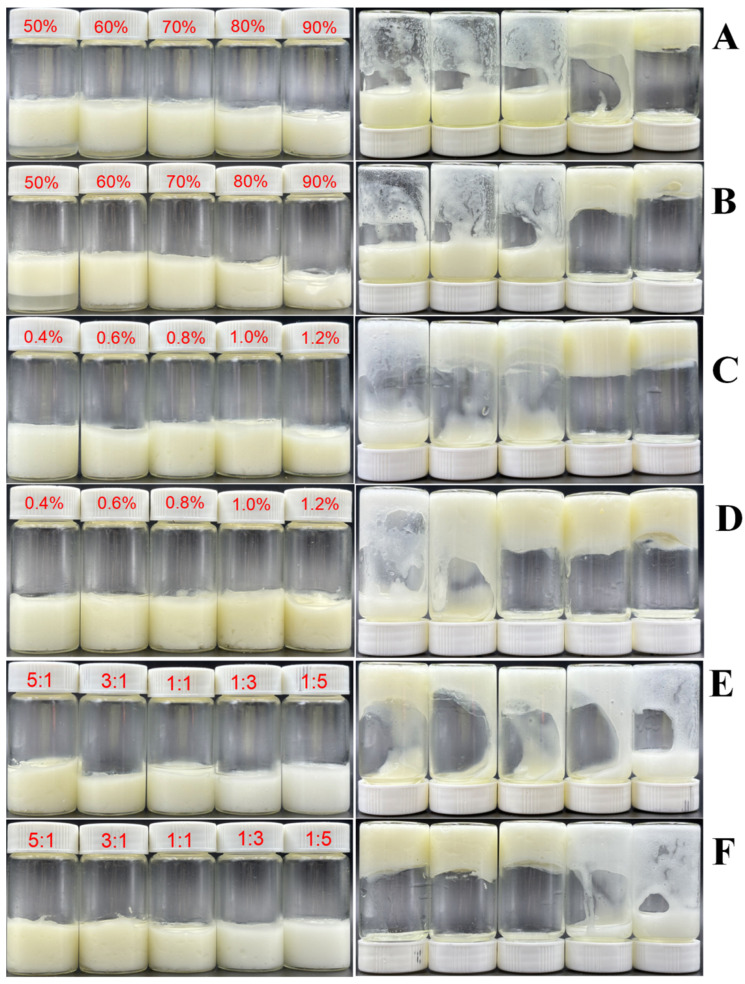
Effect of oil phase volume fraction (φ), mixture addition amount (w), and mass ratio (k) on Pickering emulsion gel formation ((**A**): φ = 50–90%, k = 1:1, w = 0.6%; (**B**): φ = 50–90%, k = 1:1, w = 1.0%; (**C**): φ = 80%, k = 1:1, w = 0.4–1.2%; (**D**): φ = 80%, k = 3:1, w = 0.4–1.2%; (**E**): φ = 80%, w = 0.6%; k = 5:1–1:5, (**F**): φ = 80%, w = 1.0%; k = 5:1–1:5).

The formation of Pickering emulsion gels was systematically examined in relation to three key factors: φ, w, and k. Figure 3 illustrates the state of the emulsion after standing at room temperature for 24 h (left side) and after being inverted for 2 h (right side). The bottle inversion method serves as a visual indicator of the efficacy of emulsion gel formation. Figure 3A,B reveal that stable emulsion gels are achieved only when φ is 80% and 90%, under varying w conditions. This finding suggests that the mixture of Hpd and LY can stabilize HIPEs, differing from the emulsions stabilized solely by Hpd particles [12]. The altered properties of the mixed compounds appear to enhance their ability to reside in the oil phase at a higher oil–water interface, thereby improving emulsification. As φ increases, the effectiveness of emulsion gel formation improves, corroborating the findings of Xiong et al. [34]. Figure 3C,D show that at a fixed φ (80%), the emulsion gel stability progressively improved with increasing w across different k values. This improvement can be attributed to inadequate interfacial particle coverage for effective Pickering stabilization at lower mixture concentrations (w < 0.8%). As the w increases, more particles occupy the oil–water interface, fully covering the oil droplet surface. This resultant thicker and stronger interfacial layer thereby enhances emulsion gel stability [35]. Figure 3E,F reveal a positive correlation between the Hpd proportion in the mixture and the efficacy of HIPE formation. When k ≤ 1:1, even increasing the addition amount (w = 1.0%) does not yield stable HIPEs. In conclusion, stable HIPEs can be formed when φ = 80%, w ≥ 0.8%, and k ≥ 1:1. Compared with protein–polyphenol HIPEs reported in the literature, such as zein–tannic acid systems requiring w ≥ 1.5% [8], our Hpd–LY mixture achieves stable gels at a lower total concentration (w = 0.8%), which is an advantage in cost and clean-label formulation. However, unlike some polysaccharide-based stabilizers that work across a wide range of flavonoids, our system is selective: only Hpd among the four tested flavonoids produced stable HIPEs, because its superior self-emulsifying ability is necessary for the synergy with LY [12]. This selectivity can be a disadvantage if one wishes to use other citrus flavonoids. Nevertheless, the broad acceptable k range (≥1:1) offers a flexible formulation.

### 3.5. Analysis of Emulsion Gel Droplet Diameter

The stability and mechanical performance of an emulsion are significantly influenced by the size of its droplets [36]. By examining the D6-D90 particle size distribution under varying conditions of φ, w, and k, the influence of these factors on droplet diameter was investigated. As presented in Table 2, increasing the w, from 0.6% to 1.2% results in a gradual decrease in droplet diameter. At lower w, particles are inadequately arranged at the oil–water interface, leading to decreased emulsion stability and facilitating droplet aggregation into larger sizes. Conversely, as the w rises, emulsion stability improves, gel formation is enhanced, and the movement and dispersibility of the oil phase diminish, thereby effectively preventing droplet aggregation. In contrast, as the φ increases, the droplet diameter also increases. At lower φ, particles effectively encapsulate oil droplets, preventing aggregation and resulting in smaller droplet sizes. However, as φ increases, the interfacial area expands, the particle adsorption layer becomes thinner, and droplet collisions and aggregations become more frequent, leading to larger particle sizes. Notably, at φ = 80%, forming a HIPEs, the droplet diameter significantly increases (*p* < 0.05). In such concentrated emulsions, the attraction between droplets intensifies, making coalescence more likely. Dai et al. [37] observed similar trends when constructing HIPEs using composite particles of β-lactoglobulin and propylene glycol alginate. Furthermore, a smaller proportion of Hpd in the mixed particles correlates with a smaller droplet diameter, contrary to the pattern observed when Hpd is used alone. This suggests that the interaction between Hpd and LY may not be a simple additive effect [12,16]. The droplet size (D50) of the Hpd–LY HIPEs is slightly larger than that of dihydromyricetin–LY HIPEs and some protein–polysaccharide systems [11,38]. This could be a disadvantage for applications requiring very fine texture. On the other hand, this system offers excellent tunability: increasing w from 0.6% to 1.2% progressively reduces droplet size, and adjusting k provides additional control. Notably, unlike Hpd alone where a higher flavonoid proportion increases droplet size [12], the Hpd–LY complex shows an opposite trend (more Hpd leads to larger droplets), indicating that LY modifies the packing behavior at the interface—a unique feature not observed in many protein–flavonoid systems.

### 3.6. Formation of O/W Emulsion

**Figure 4:**
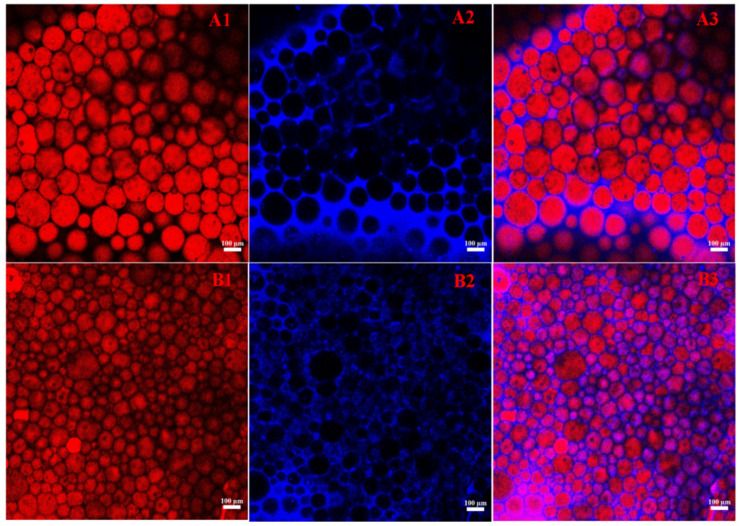
Confocal laser images of Pickering HIPEs with Hpd stabilization at different w and k ((**A1**–**A3**): φ = 80%, w = 1.0%, k = 5:1; (**B1**–**B3**): φ = 80%, w = 1.2%, k = 3:1) (Both Hpd and LY stained by Nile Blue A appeared blue while the oil phase treated with Nile Red exhibited red regions).

Laser confocal microscopy is widely employed to obtain high-resolution images of emulsion structures, facilitating precise visualization of the distribution of solid particles and oil droplets within the emulsion. This technology significantly enhances the understanding of the internal phase structure of emulsions, the arrangement of particles, and their influence on emulsion stability [39]. As illustrated in Figure 4, the blue regions denote the oil phase, while the red regions indicate the mixed particles. This staining method allows for a clear observation of the emulsion’s organizational structure, enabling further analysis of the emulsion type. The emulsion observed in this experiment is classified as an O/W emulsion. Notably, this system is an aggregated emulsion gel: the gel-like properties arise from a dense interfacial layer, inter-droplet crosslinking, and restricted droplet movement, rather than from continuous phase gelation. Therefore, the CLSM images show typical O/W droplets, while the whole system exhibits macroscopically measurable gel characteristics. Figure 4(A1–A3) and Figure 4(B1–B3) depict the emulsion structures under varying conditions of w and k, respectively. The images clearly demonstrate that as w increases and the proportion of Hpd decreases, the diameter of the oil droplets in the emulsion progressively reduces, and the arrangement of the oil droplets becomes more compact. This observation aligns with the analysis of droplet diameter variation, suggesting that appropriate control of w and k enhances emulsion stability and promotes a more uniform droplet dispersion. This finding corroborates the notion that adjusting formulation parameters can effectively improve the physical properties and stability of the emulsion. Chen et al. also reported that the microstructure of Pickering emulsions was closely related to the concentration of stabilizing particles and the oil phase fraction. With increasing oil phase fraction, the spaces between oil droplets decreased and a more compact emulsion network was formed, thereby improving the stability of the emulsion [40].

### 3.7. Textural Analysis of HIPEs

**Figure 5:**
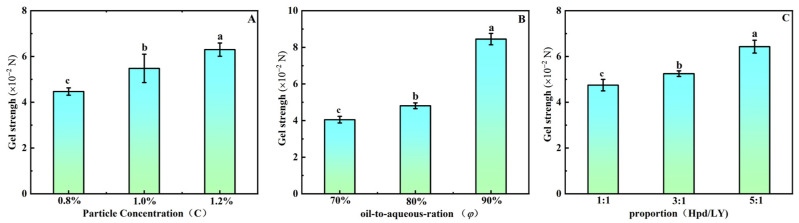
The effect of w (**A**), φ (**B**), and k (**C**) on the gel strength of HIPEs. (The letters a, b, c denote significant differences (*p* < 0.05) among the samples prepared under the indicated conditions.).

This study, as illustrated in Figure 5, investigates the influence of three variables—φ, w, and k—on the gel strength of emulsions. With φ fixed at 80% and k = 3:1, an increase in the mixture w leads to a significant enhancement in gel strength (*p* < 0.05). This enhancement can be explained by the strengthened attachment of composite particles at the oil–water interface, enabling tighter packing into a robust interfacial layer that significantly enhances gel rigidity. These results are consistent with the work of Jing et al. [41], which established a direct correlation between interfacial particle packing density and the resultant gel characteristics in emulsions. Furthermore, higher φ values intensified oil droplet collisions and coalescence within the emulsion, leading to enlarged droplet dimensions and more compact droplet packing. This alteration further fortifies the gel network, implying that larger oil droplets contribute to the enhanced stability of the gel structure [42]. In Section 3.5, we observed that the proportion of hesperidin (Hpd) in the mixture correlates positively with the diameter of emulsion droplets, providing insight into the gradual strengthening of gel structure as the k increases. Specifically, as k rises, the particle distribution within the emulsion becomes more uniform, further reinforcing the gel structure. In conclusion, increases in φ, w, and k positively affect the enhancement of emulsion gel strength. By optimizing these parameters, the stability of the emulsion can be significantly improved, offering a theoretical foundation for its application. The gel strength of the HIPEs is comparable to soy protein isolate-based HIPEs used as fat replacers [43]. A key advantage is the steep response to w and k: small increases in Hpd proportion or total concentration yield substantial gel strengthening, enabling precise texture engineering with minimal ingredient usage. However, compared to covalently cross-linked particle systems, the physically assembled complex has lower maximum gel strength at equivalent particle concentration. At very high k, the gel becomes quite firm, which may be too brittle for spreadable products—a potential disadvantage that requires case-by-case optimization.

### 3.8. Analysis of the Protective Effects of HIPEs on Lutein

**Figure 6:**
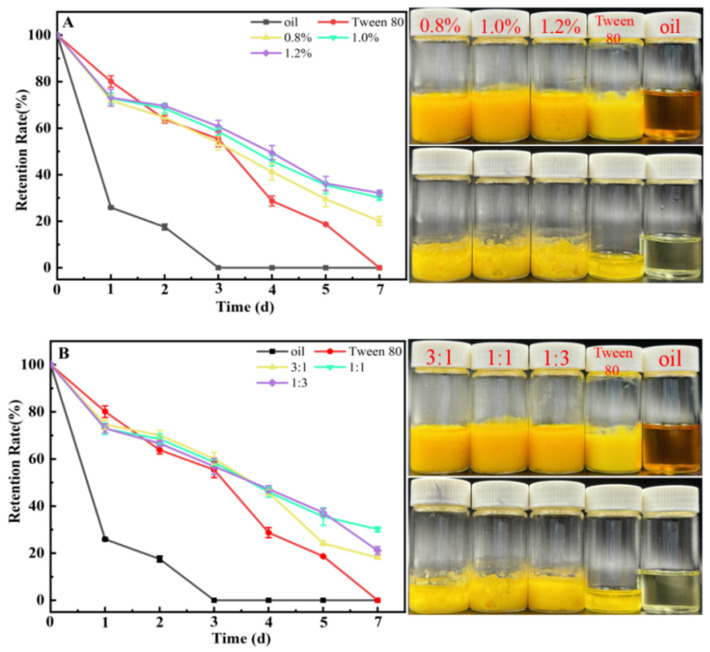
The effect of w (**A**) and k (**B**) on the UV protection of lutein by Pickering HIPEs.

Free lutein is highly sensitive to temperature and light, making it susceptible to irreversible oxidation and degradation. Consequently, developing methods to enhance lutein’s stability is of significant importance [44]. Zhou et al. [45] demonstrated that curcumin degradation in emulsions could be effectively prevented by employing zein/pectin hybrid particles to form HIPEs. Similarly, Geng et al. [16] utilized a dihydromyricetin/lysozyme mixture to construct Pickering HIPEs, which successfully protected lutein from ultraviolet (UV) light-induced degradation. Figure 6 illustrates the efficacy of Pickering HIPEs at various mixture concentrations and mass ratios in preventing UV-induced lutein degradation at 37 °C, using a control composed of a lutein-containing oil phase and Tween 80. The findings reveal that Pickering HIPEs formulated with the Hpd/LY mixture significantly improved lutein’s resistance to UV exposure compared to the control group. After two days of UV exposure, the lutein in the control group degraded rapidly, with its retention rate plummeting to approximately 20%. In contrast, the retention rate of lutein within the Pickering HIPEs, across various concentrations and mass ratios, consistently exceeded 60%. Over time, the effect of concentration on lutein retention indicated that the retention rate was lowest at a concentration of w = 0.8%, while the rates at w = 1.0% and 1.2% were comparable (Figure 6A). This suggests a positive correlation between mixture concentration and lutein retention rate, although the protective effect stabilizes once the concentration reaches a threshold of w = 1.0%. This stabilization may be attributed to the increased thickness of the oil droplet’s outer layer, which enhances UV blocking, and the antioxidant properties of hesperidin, which further inhibit lutein degradation. Figure 6B demonstrates that Pickering HIPEs constructed with different mass ratios effectively inhibit lutein degradation, although variations in efficacy among different k values are minimal.

### 3.9. Analysis of the Resistance of HIPEs to Lipid Oxidation

**Figure 7:**
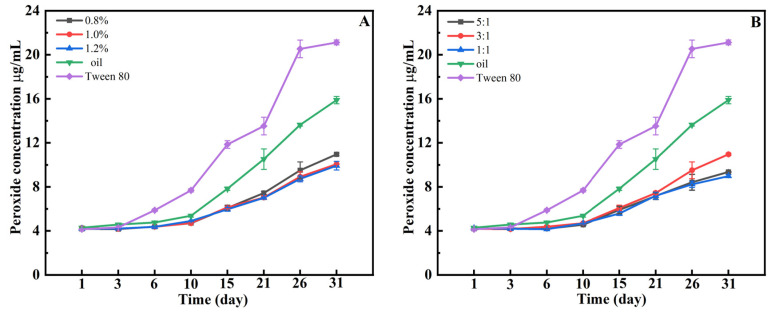
The effect of w (**A**) and k (**B**) on peroxide content in Pickering HIPEs.

The oxidation rate of oils in emulsions generally exceeds that of pure oils [46]. In HIPEs, the elevated oil phase volume fraction contributes to a heightened susceptibility to oxidation. Plant polyphenols, renowned for their potent antioxidant properties, can form complexes with proteins or polysaccharides, thereby creating a denser interfacial layer that mitigates lipid oxidation in Pickering emulsions [47]. This study investigated the variations in lipid oxidation product content in HIPEs formulated under diverse w and k conditions over a period of 31 d. As depicted in Figure 7, at 37 °C, the peroxide content in emulsions prepared with sunflower oil and Tween 80 exhibited a significant increase after 10 d, reaching 16 μg/g and 21 μg/g, respectively, after 31 d. The rapid oxidation rate of oils in emulsions containing Tween 80 corroborates the assertion that oil oxidation in emulsions generally surpasses that in pure oils. Conversely, in HIPEs formulated with w and k, the increase in peroxide content was slower and consistently lower than that of the control group, indicating that the mixture effectively inhibited oil oxidation. This difference in oxidative stability can be attributed to two complementary mechanisms of the Hpd-LY complex. The first is radical scavenging: hesperidin possesses phenolic hydroxyl groups that donate hydrogen atoms or electrons to quench free radicals, thereby terminating lipid oxidation chain reactions. The second is an interfacial barrier: the dense layer of solid Hpd-LY particles adsorbed at the oil–water interface physically limits the diffusion of pro-oxidants (e.g., oxygen, metal ions) from the aqueous phase to the oil core and restricts the outward migration of lipid radicals. In contrast, Tween 80 lacks intrinsic radical-scavenging groups and forms a thin, dynamic interfacial layer that provides poor barrier protection. Moreover, Tween 80 is known to undergo autoxidation, potentially generating pro-oxidant species [48,49]. These combined effects explain why Hpd-LY-stabilized HIPEs outperform Tween 80-stabilized emulsions in retarding lipid oxidation. A similar antioxidant effect was observed by Zeng et al., who reported that HIPEs constructed with alcohol-soluble protein/chitosan mixed particles similarly decelerated lipid oxidation reactions [50]. After 31 d, the peroxide value of our HIPEs remained below ~8 μg/g, while Tween-80 emulsions exceeded 20 μg/g. This performance is better than many tea polyphenol–protein systems [47]. The advantage is that Hpd is positioned directly at the oil–water interface as part of the emulsifier, providing both a radical-scavenging and a physical barrier effect. However, a disadvantage is that the protection depends on a sufficient Hpd proportion (k ≥ 1:1), which increases cost and potential bitterness. Compared to synthetic antioxidants like BHA, the natural hesperidin is less potent on a molar basis, but the interfacial localization compensates for this drawback.

## 4. Conclusions

This study demonstrates that among the four citrus flavonoids tested (Hpt, Hpd, Neohpd, and Neohpddic), the complex formed between Hpd and LY exhibits a superior ability to form stable HIPEs. The Hpd-LY complex showed an increased three-phase contact angle compared to Hpd alone, which facilitated the direct formation of HIPEs. The formation and properties of these HIPEs were critically influenced by the φ, w, and k. Stable HIPEs were achieved under the conditions of φ = 80%, w ≥ 0.8%, and k ≥ 1:1. The average droplet diameter decreased with increasing w but increased with higher φ and k. Concurrently, the mechanical strength of the HIPEs was enhanced by increasing these three parameters. Moreover, the Hpd-LY-stabilized HIPEs offered excellent protection for encapsulated lutein against UV degradation and effectively retarded lipid oxidation. This research not only elucidates the formation mechanism of flavonoid–protein-based HIPEs but also provides a foundation for designing stable, functional emulsion systems for food and pharmaceutical applications. However, we also recognize some limitations, including sensory aspects such as the potential bitterness of hesperidin and changes in mouthfeel, scalability from lab scale to industrial production, and compatibility with complex food matrices containing salts, sugars, or other ingredients. Future studies should therefore test these Hpd-LY HIPEs in real food products like beverages, dairy products, or sauces, evaluate their sensory properties through panel testing, and explore large-scale preparation methods. In vivo digestion, absorption, and bioavailability of the encapsulated lutein also need to be investigated before practical applications can be considered.

## Figures and Tables

**Table 1:** Three-phase contact angles (θ) of LY, Hpd, and Hpd/LY (1:1) particles.

Name	LY	Hpd	Hpd/LY
Contact angles (θ)	80.52° ± 0.44	51.16° ± 0.58	77.04° ± 0.89

**Table 2:** The effect of w, φ and k on droplet diameter of emulsion.

k	φ (%)	w (%)	D_6_ (μm)	D_10_ (μm)	D_25_ (μm)	D_50_ (μm)	D_75_ (μm)	D_90_ (μm)
3:1	80	0.6	5.12 ± 0.46 ^o^	14.16 ± 1.74 ^m^	47.69 ± 1.54 ^j^	75.32 ± 2.28 ^g^	106.30 ± 3.55 ^d^	140.20 ± 4.25 ^a^
		0.8	4.67 ± 0.11 ^o^	13.57 ± 1.49 ^m^	47.99 ± 0.97 ^j^	72.93 ± 0.83 ^g^	99.61 ± 1.37 ^e^	128.86 ± 3.84 ^b^
		1.0	4.49 ± 0.15 ^o^	13.36 ± 0.89 ^m^	43.66 ± 1.23 ^k^	65.97 ± 1.61 ^h^	89.33 ± 2.66 ^f^	113.56 ± 7.60 ^c^
		1.2	3.92 ± 0.17 ^o^	9.85 ± 0.60 ^n^	38.70 ± 0.55 ^L^	57.77 ± 0.56 ^i^	75.77 ± 0.52 ^g^	91.75 ± 0.29 ^f^
**k**	**w (%)**	**φ (%)**	**D_6_ (μm)**	**D_10_ (μm)**	**D_25_ (μm)**	**D_50_ (μm)**	**D_75_ (μm)**	**D_90_ (μm)**
3:1	0.8	50	5.00 ± 0.47 ^m^	14.72 ± 1.75 ^L^	40.43 ± 1.90 ^k^	60.80 ± 2.27 ^i^	81.24 ± 3.69 ^f^	102.71 ± 8.39 ^d^
		60	5.59 ± 0.15 ^m^	17.38 ± 0.70 ^L^	45.06 ± 1.15 ^jk^	66.00 ± 1.48 ^hi^	87.82 ± 2.04 ^e^	108.83 ± 2.81 ^c^
		70	4.81 ± 0.20 ^m^	15.84 ± 1.04 ^L^	45.80 ± 0.49 ^jk^	68.22 ± 1.00 ^gh^	91.89 ± 1.77 ^e^	116.80 ± 3.50 ^b^
		80	4.68 ± 0.11 ^m^	13.57 ± 1.49 ^L^	47.99 ± 0.98 ^j^	72.93 ± 0.84 ^g^	99.82 ± 1.52 ^d^	128.87 ± 3.48 ^a^
**φ (%)**	**w (%)**	**k**	**D_6_ (μm)**	**D_10_ (μm)**	**D_25_ (μm)**	**D_50_ (μm)**	**D_75_ (μm)**	**D_90_ (μm)**
80	0.6	5:1	26.28 ± 0.58 ^o^	34.13 ± 0.29 ^m^	57.29 ± 0.56 ^j^	82.88 ± 0.67 ^f^	111.97 ± 0.50 ^c^	137.30 ± 0.10 ^a^
		3:1	23.82 ± 1.29 ^p^	30.81 ± 1.71 ^n^	54.74 ± 1.45 ^k^	77.60 ± 1.51 ^g^	100.21 ± 1.75 ^d^	118.73 ± 2.42 ^b^
		1:1	18.88 ± 1.68 ^q^	24.18 ± 1.33 ^op^	44.22 ± 1.96 ^L^	63.78 ± 0.94 ^i^	80.96 ± 0.69 ^k^	95.30 ± 1.46 ^e^
		1:3	16.99 ± 1.41 ^q^	22.92 ± 1.26 ^p^	36.25 ± 2.06 ^m^	56.88 ± 1.50 ^jk^	70.71 ± 0.43 ^h^	82.60 ± 0.82 ^f^

Note: Data are presented as three independent groups. Within each group, different lowercase letters denote statistically significant differences (*p* < 0.05).

## Data Availability

The data presented in this study are available on request from the corresponding author due to intellectual property protection.
